# Herbivore consumers face different challenges along opposite sides of the stoichiometric knife‐edge

**DOI:** 10.1111/ele.13386

**Published:** 2019-09-11

**Authors:** Libin Zhou, Steven A. J. Declerck

**Affiliations:** ^1^ Department of Aquatic Ecology Netherlands Institute of Ecology (NIOO‐KNAW) Wageningen the Netherlands

**Keywords:** *Brachionus calyciflorus*, carbon to phosphorus ratio, compensatory feeding, ecological stoichiometry, elemental homeostasis, homeostatic breakdown, phosphorus metabolism, rotifera, zooplankton

## Abstract

Anthropogenic activities have reshaped the relative supply rates of essential elements to organisms. Recent studies suggested that consumer performance is strongly reduced by food that is either very high or very low in relative phosphorus content. However, the generality of such ‘stoichiometric knife‐edge’ and its underlying mechanisms are poorly understood. We studied the response of a planktonic rotifer to a 10‐fold food carbon : phosphorus (C : P) gradient and confirmed the existence of the stoichiometric knife‐edge. Interestingly, we observed a complete homeostatic breakdown associated with strong growth reductions at high food C : P. In contrast, at low food C : P, animals maintained homeostasis despite pronounced performance reductions. Our results suggest that the mechanisms underlying adverse effects of stoichiometric imbalance are determined by both the identity of elements that are limiting and those that are present in excess. Negative effects of excess P reveal an additional way of how eutrophication may affect consumers.

## Introduction

In recent decades, anthropogenic activities have strongly altered the relative supply rates of key elements, such as carbon (C), nitrogen (N) and phosphorus (P) to organisms (Smith & Schindler 2009; Elser *et al.* 2009). The elemental composition of autotrophs is known to be different and more flexible than that of their consumers (Sterner & Elser 2002; Persson *et al.* 2010; Van de Waal *et al.* 2010). Mismatches of elemental ratios between producers and consumers may have negative effects on consumer performance (Elser *et al.* 2001; Hessen *et al.* 2013; Wagner *et al.* 2013), trophic transfer efficiency (Grover 2003; Rowland *et al.* 2015) and the strength of top‐down control (Hall 2009; Declerck *et al.* 2015). To understand how altered elemental supply rates affect food web structure and ecosystem functioning, it is important to know when stoichiometric mismatch occurs, how it affects consumers and how they respond accordingly.

Generally, the growth of organisms is thought to be constrained by the nutrient that is relatively in the shortest supply (Liebig’s law of minimum; Liebig 1840; Hessen *et al.* 2013). Accordingly, a well‐developed concept in ecological stoichiometry (ES) is the threshold elemental ratio (TER; Urabe & Watanabe 1992; Frost & Elser 2002; Anderson & Hessen 2005; Frost *et al.* 2006), which represents the ratio of two elements at which the identity of the limiting element shifts from one to the other element. TER predicts a unimodal response of consumer performance to a gradient of food elemental ratios, with optimal growth close to the requirements of the organisms (TER) and a reduction towards the extremes of the gradient (Anderson & Hessen 2005; Frost *et al.* 2006; Khattak *et al.* 2018). This prediction has been supported for a wide range of taxa (coined as the ‘stoichiometric knife‐edge’; Elser *et al.* 2006; Bullejos *et al.* 2014; Benstead *et al.* 2014; Laspoumaderes *et al.* 2015; Elser *et al.* 2016). However, most of these studies attribute the performance reduction of consumers fed low C : P food more to disadvantages associated with excess P than to C limitation (Boersma & Elser 2006; Hessen *et al.* 2013). For example, storage (Persson *et al.* 2010) or the elimination of excess P (Anderson *et al.* 2005; Boersma & Elser 2006; Elser *et al.* 2006) is thought to be highly costly, and to be associated with a growth penalty in heterotrophs. P may itself also be toxic to animals, for example, because of its harmful effects on symporter functions of gut epithelial cells (Karasov & Martinez del Rio 2007). Alternatively, Plath & Boersma (2001) suggested that a low food C : P ratio may indirectly result in C limitation of *Daphnia* because they adjust their feeding rates in function of their P intake.

Another key concept in ES is elemental homeostasis, which reflects the ability of an organism to keep its somatic elemental composition constant in the face of varying elemental supply ratios (Elser & Urabe 1999; Sterner & Elser 2002). In contrast to autotrophs, animal consumers have long been considered as being very homeostatic (Andersen & Hessen 1991; Sterner & Elser 2002; Anderson *et al.* 2005). However, evidence for considerable plasticity in consumer body stoichiometry has recently accumulated for a wide range of organisms (Small & Pringle 2010; Prater *et al.* 2017; Teurlincx *et al.* 2017), raising the notion that organisms may take different positions along a continuum between strict regulators and strict conformers (Persson *et al.* 2010; Meunier *et al.* 2014). Most work on consumer homeostasis assumes that the somatic elemental composition of consumers responds proportionally to changes in the elemental composition of its food source and that the strength of this response is constant across the whole stoichiometric food quality range (Sterner & Elser 2002; Persson *et al.* 2010; Hessen *et al.* 2013). However, Meunier *et al. *(2014) introduced a more physiology‐oriented view on the concept and questioned the linearity of this response. Indeed, homeostasis is maintained by the simultaneous action of multiple regulating processes (Frost *et al.* 2005; He & Wang 2008; Hessen *et al.* 2013), such as feeding (Suzuki‐Ohno *et al.* 2012; Urabe *et al.* 2018), assimilation (DeMott *et al.* 1998; Urabe *et al.* 2018), allocation (Urabe & Sterner 2001; Frost *et al.* 2010), excretion (Frost *et al.* 2004, 2005) and respiration (Jensen *et al.* 2006; Hessen & Anderson 2008). Variation in the efficiency of these processes along a stoichiometric food gradient is very likely and is expected to result in a variable homeostatic strength across such gradient. Meunier *et al. *(2014) also suggested that conformers and regulators should differ in the shape of their stoichiometric response curves. Lack of suitable datasets has so far prevented further confirmation of these expectations.

Variation in degree of homeostasis likely reflects different adaptive strategies of organisms; however, benefits and disadvantages of homeostasis are still poorly understood (Persson *et al.* 2010; Hessen *et al.* 2013; Leal *et al.* 2017). Organisms require various biomolecules (e.g. proteins, nucleic acids, lipids) in specific ratios (Elser *et al.* 1996). At optimal growth, somatic elemental composition reflects the ideal ratios of these biomolecules and their elemental make‐up (Sterner & Elser 2002; Franklin *et al.* 2011; Manzoni *et al.* 2017). By maintaining elemental homeostasis, regulators strive towards the maintenance of a specific somatic elemental composition to secure optimal functioning. The disadvantage of this strategy is that as food elemental composition deviates from this optimum, growth will be limited by the element in shortest supply while excess elements need to be eliminated. Such strategy will thus inevitably result in a reduced resource exploitation efficiency (Anderson *et al.* 2005; Frost *et al.* 2005; Manzoni *et al.* 2017). In contrast, conformers have the ability to track the elemental composition of their food. Such relaxation of homeostasis may potentially be achieved without relevant performance reductions if excess elements can be stored without costs (Persson *et al.* 2010; Hood & Sterner 2010) or if the utilisation efficiency of the limiting element can be increased (Jeyasingh *et al.* 2009; Prater *et al.* 2017). Alternatively, however, stoichiometric plasticity may also reflect an inability to cope with stoichiometric imbalance. For example, the capacity of regulators to maintain homeostasis has likely its limits. Once the degree of stoichiometric mismatch trespasses these limits, we expect abrupt deviations in somatic elemental composition to coincide with strong reductions in performance.

A question that so far has largely remained unaddressed is to what extent responses of organisms to a broad food stoichiometry gradient are consistent along the opposite sides of their growth optimum. Studies on the stoichiometric knife‐edge (Boersma & Elser 2006; Elser *et al.* 2006; Elser *et al.* 2016) implicitly suggest that organisms face different challenges when being confronted to opposing directions of stoichiometric mismatch, that is, costs associated with P limitation and excess C at high C : P vs. costs associated with excess P at low C : P conditions. Currently, we do not know much yet about how organisms respond to these different challenges and whether coping strategies also involve different levels of homeostatic strength. Such knowledge is nevertheless a prerequisite to better understand and predict the direct consequences of stoichiometric mismatch for the role of consumers in ecosystem functions such as nutrient cycling (Elser & Urabe 1999; Atkinson *et al.* 2017) and the trophic transfer of energy and materials (Boersma *et al.* 2008; Rowland *et al.* 2015), not only when nutrients are limiting but also under conditions of severe anthropogenic eutrophication.

The experimental exposure of organisms to broad gradients of food elemental composition allows studying the association of organism performance with variation in their somatic elemental composition. This may not only help the interpretation of deviations from homeostasis but also cast a light on the costs and benefits from contrasting strategies with which organisms respond to stoichiometric mismatch. For this study, we subjected a metazoan planktonic consumer, that is, the monogononta rotifer *Brachionus calyciflorus* to a 10‐fold gradient of food C : P ratios and measured the response of two measures of performance (somatic and population growth rate), somatic elemental composition and two key regulating physiological functions (i.e. food uptake and P‐loss rates). First, we wanted to study the consumer performance response to a broad food C : P range and test for the existence of a stoichiometric knife‐edge. Second, given that organisms may face different challenges along opposite directions of a stoichiometric food gradient, we assessed variation in the degree and modes of stoichiometric regulation in response to stoichiometric imbalance at both sides of the growth optimum (i.e. towards very high and very low resource C : P). Third, by relating consumer performance with the degree of stoichiometric plasticity along each of these directions, we evaluated to what extent homeostatic regulation implies reduced performance, and whether an apparent relaxation of such regulation reflects a capacity to deal with stoichiometric mismatch or, alternatively, an inability to maintain elemental homeostasis. Finally, we discussed the potential implications of these results for real‐world systems.

## Methods and Materials

### Rotifer and algae cultures

The *B. calyciflorus* clone used in our study was obtained from the dormant egg bank of a Dutch lake (52°5′26.50″N; 4°20′18.40″E). To exclude the influence of indirect effects of P limitation on the nutritional quality of algae (Zhou *et al.* 2018), we created a stoichiometric food quality gradient by enriching P‐limited chemostat‐grown green algae (*Chlamydomonas reinhardtii*) with different concentrations of P shortly before feeding to the rotifers (see Appendix S1). In this way, we created nine food quality treatments representing a 10‐fold food C : P gradient ranging from 53 to 583. This gradient was chosen to impose a strong stoichiometric mismatch between producers and consumers via both high and low food C : P values while spanning the range of conditions found in nature (Hessen 2006; Sterner *et al.* 2008).

### Population growth rate

The response of rotifer exponential population growth rate to the respective food quality treatments was studied in flasks filled with a 200 mL algal food suspension at *ad libitum* concentrations (1000 μmol C L^−1^), 24 °C and continuous darkness. We kept the cultures in an exponential growth by daily restarting populations with a subsample of approximately 4000 individuals in a fresh food suspension (see Appendix S1). Exponential population growth rate was repeatedly calculated as (lnN_t_ − lnN_0_)/t on a daily basis, where N_0_ and N_t_ represent the population size at the start and end of each 24‐h period, respectively. Population growth rate for each treatment was calculated as the mean of 12 consecutive 24‐h periods.

### Somatic growth rate

For somatic growth rate, we collected a cohort of 100 newborns (age < 2 h) from the populations of the population growth rate experiment, incubated them for 18 h (t) in the respective food quality treatments and measured their total C (M_t_). We also measured the carbon content of groups of 100 neonates born in the respective food quality treatments (M_0_; see Appendix S1). Mass‐specific somatic growth rate was calculated as (lnM_t_ – lnM_0_)/t.

### Food ingestion, P‐intake and P‐loss rate

For the measurement of food ingestion, P‐intake and P‐loss rates, we used animals from the population growth rate experiment to ensure that animals were physiologically adapted to different food qualities. All rates were measured on the same sets of animals. For the ingestion rate experiment, we allowed groups of 200 rotifers to feed for 4 h on algae suspensions of the different food quality treatments (see Appendix S1). Ingestion rates were calculated as (C_t_ − C_0_) v/(nt) (Peters 1984), where C_t_ and C_0_ represent the final and initial algal C concentration, respectively, in each vial, v is the volume of food suspension in each vial (8 mL), n is the rotifer number and t is the time period of incubation. Algal C concentrations were calculated by estimating algal biovolume using a coulter counter (Multisizer‐tm 3 Coulter Counter, Beckman Coulter). These biovolumes were subsequently converted into molar concentrations of C using a previously established biovolume‐carbon regression equation. P‐intake rate was estimated from C ingestion rate and the respective algal elemental composition in each food quality treatment (Oftedal 1991).

For the measurement of P‐loss rates, we transferred the rotifers from the grazing experiments into 1.5 mL volumes of nutrient‐free WC medium (Guillard & Lorenzen 1972). After an incubation of 4 h in the dark on a rotating plankton wheel (30 rpm), the medium from each unit was filtered through a 30‐µm mesh to remove rotifers and transferred into a 1.5‐mL glass vial. After being autoclaved for 30 min under 121 °C, the samples were stored for the later measurement of dissolved P (i.e. orthophosphates). P‐loss rates were estimated as (*Pv*)/(*nt*), where *P* refers to sample P content, *v* and *t* represent sample volume and incubation time, respectively, *n* is the rotifer number in each treatment.

### Stoichiometric measurements

During the experiment, we measured algal C, N, P content of each food quality treatment at three occasions. For the measurement of rotifer elemental content, we used animals from the population growth rate experiment. From each culture, we incubated two samples of 150 females each carrying one parthenogenetic egg in nutrient‐free WC medium for 30 min to allow evacuation of the guts. C and N content of algae and rotifer samples were determined using a FLASH 2000 organic element analyzer (Interscience B.V., Breda, Netherlands). For P, the algal and rotifer samples were first incinerated at 550 °C for 45 min and autoclaved in 2.5% potassium persulfate (K_2_S_2_O_8_) solutions at 121 °C for 30 min. These samples and the samples from the P‐loss rate experiment were measured using a QuAAtro segmented flow autoanalyzer (Beun de Ronde, Abcoude, the Netherlands). All stoichiometric ratios are expressed as molar ratios.

### Data analysis

The responses of rotifer growth rates, somatic elemental ratios, food ingestion rates, and P‐intake and P‐loss rates to diet C : P were statistically evaluated by contrasting three alternative models, that is, a non‐quadratic linear model, a quadratic linear model and a piecewise regression model. Models were ranked based on the Akaike Information Criterion (AIC). Generally, the model with the lowest AIC value was used for interpretation. However, if alternative models proved equally good (i.e. with ∆AIC < approximately 2; Burnham & Anderson 2004), they were chosen to maximise comparability across analyses. Where relevant, quadratic and piecewise regression models were used to calculate the location of the optima of unimodal responses. Elemental ratios, P‐intake and P‐loss rates were log_2_‐transformed prior to analysis. All the statistical analyses were performed in R (R Core Team, 2016). The piecewise regression was conducted using the package ‘segmented’, and the Davies test provided by this package was applied to determine the significance of differences between slopes (Muggeo 2008).

## Results

The addition of P to P‐limited algae strongly increased their P content and reduced their C : P ratios (ranging from 53 to 583), but did not change their N content and C : N ratios (Appendix S2 Figs S1 and S2a–b).

**Figure 1:**
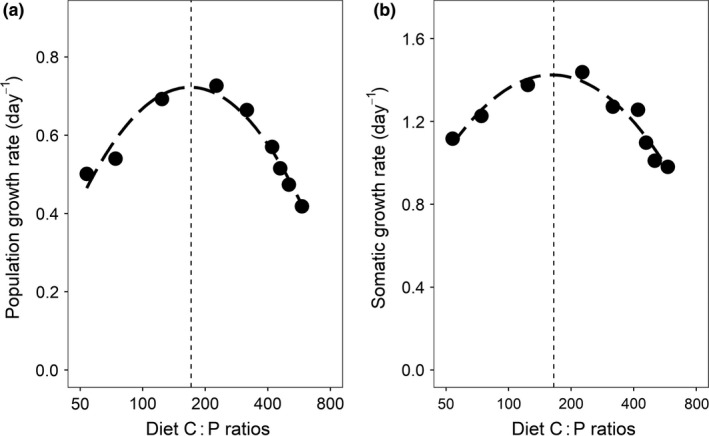
Response of rotifer population growth rate (a) and mass‐specific somatic growth rate (b) to the experimental food C : P gradient. The dashed lines represent the expected values according to the quadratic regression models; the vertical dotted lines represent the food C : P ratio corresponding to the maximal growth rate values as calculated by the quadratic regression models. Symbols in (a) represent means of values from 12 subsequent 24‐h time intervals, and symbols in (b) are the means of three technical replicates. Note the log‐scale of the X‐axes.

Rotifer population growth rate showed a unimodal response to diet C : P ratio with a maximum at intermediate diet C : P values and strong reductions towards the extremes of the resource gradient (Fig. 1a; Appendix S2 Tables S1 and S2). Using the quadratic regression model, the maximum population growth rate was estimated at a diet C : P ratio of 171. Mass‐specific somatic growth rate showed a very similar response (Fig. 1b; see Appendix S2 Table S1) with a maximum at an estimated diet C : P ratio of 165.

**Figure 2:**
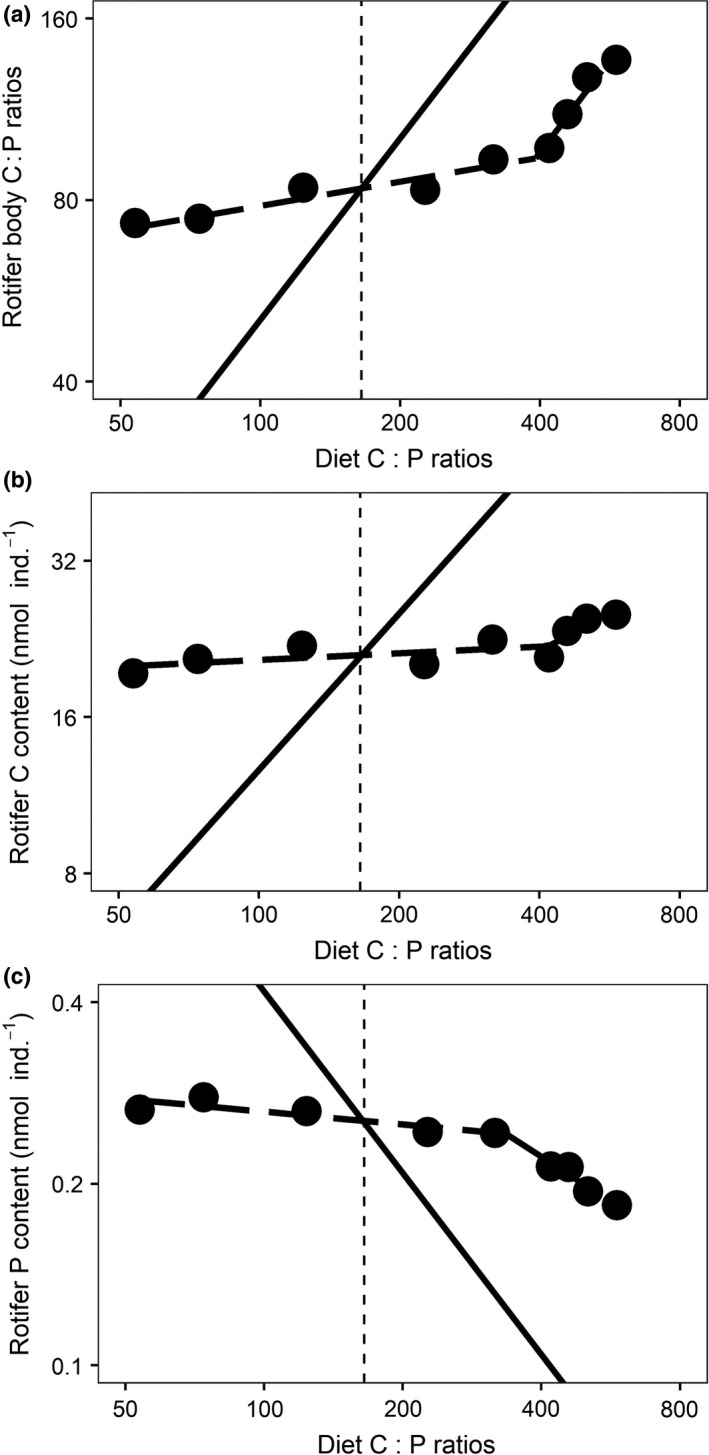
Response of rotifer stoichiometry to the experimental food C : P gradient. (a) Rotifer C : P ratio, (b) rotifer C content and (c) rotifer P content. The vertical dotted lines represent the optimal food C : P at which rotifer somatic growth was estimated to be maximal. In each figure, the solid line represents a 1 : 1 proportional change between diet C : P and the respective variables. Symbols are the means of three repeated measurements in time. Note the log‐scale of the axes.

The relationship between rotifer C : P and food C : P was best described by a two‐segment piecewise regression model and its breakpoint was situated at a food C : P of 391 (Fig. 2a, Appendix S2 Table S2). Above the breakpoint, the slope approximated 1.02, showing that rotifer C : P followed changes in food C : P ratio proportionally. In contrast, the slope of the regression equation below the breakpoint was much lower (0.125) but still differed significantly from zero (Fig. 2a; see Appendix S2 Table S2). Although the C : P response above the breakpoint coincided with an increased rotifer C content, it was mainly driven by a reduction in rotifer P content (Fig. 2b and c; Appendix S2 Table S4). Rotifer C : N ratios increased slightly along the C : P gradient mainly due to an increased rotifer C content (Appendix S2 Fig. S3).

**Figure 3:**
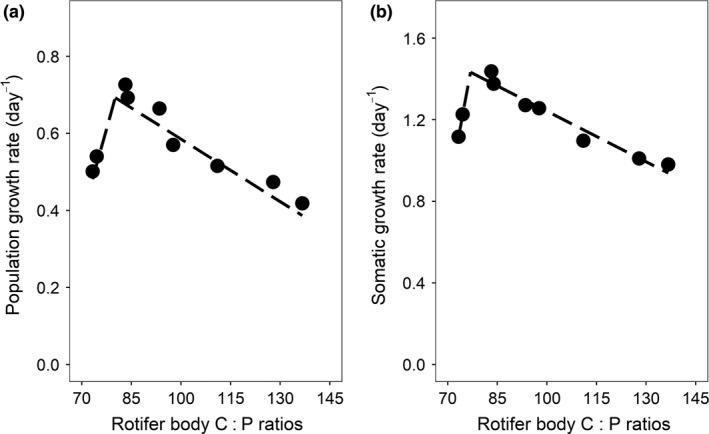
Association between rotifer population growth rate (a) and rotifer somatic growth rate (b) with rotifer C : P ratio. Symbols in (a) represent the means of values  across 12 subsequent 24‐h time intervals, and symbols in (b) are the means of three technical replicates.

According to piecewise regression (Appendix S2 Table S2), maximum population growth rate coincided with a rotifer somatic C : P ratio of 80 (Fig. 3a). Similarly, somatic maximum growth rate reached at a rotifer  C : P ratio of 77 (Fig. 3b; Appendix S2 Table S2).

**Figure 4:**
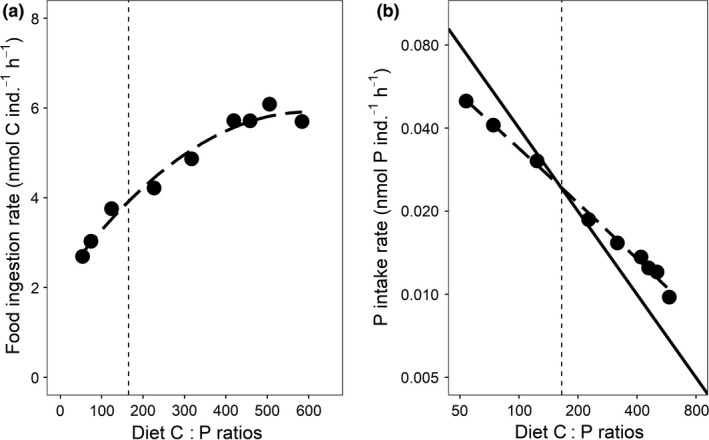
Response of rotifer food ingestion (a) and P‐intake (b) rates to a gradient in food C : P ratios. The vertical dashed lines represent the optimal food C : P at which rotifers showed the highest somatic growth rates. The solid line in Fig. 4b represents a 1 : 1 proportional change between diet C : P ratio and P‐intake rate. Symbols are the mean values across three repeated measurements in time. Note the log‐scale of the X‐ and Y‐axes in Fig. 4b.

**Figure 5:**
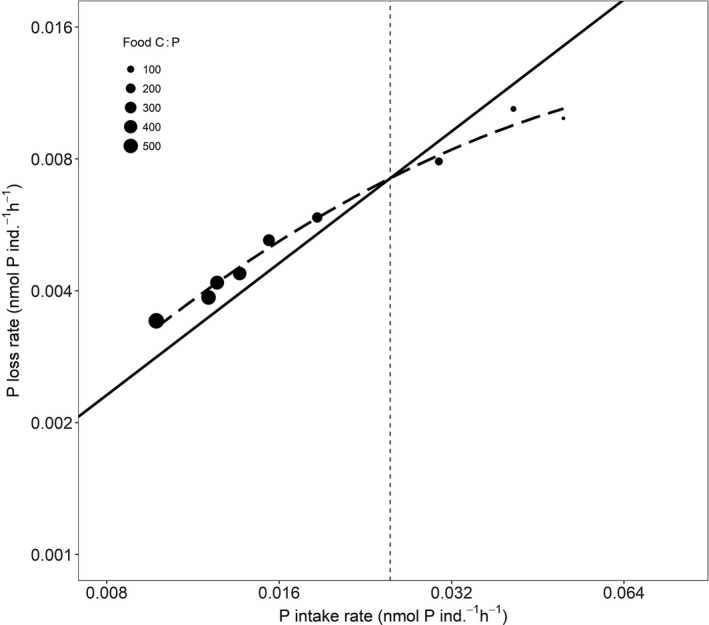
Association between rotifer P‐loss rates and P‐intake rates. The vertical dashed line represents the optimal food C : P at which rotifers showed the highest somatic growth rates. The solid line represents a 1 : 1 proportional change between the two variables. Symbols represent the averages of P‐loss rates of three repeated measurements in time. Symbol size indicates food C : P ratio. Note the log‐scale of the axes.

Both food ingestion and corresponding P‐intake rates were strongly affected by food quality (Fig. 4a–b; Appendix S2 Table S1–S3). Below the food C : P growth optimum, food ingestion rates decreased with decreasing food C : P ratios (Fig. 4a) while P‐intake rates increased but less than predicted by the 1 : 1 line (Fig. 4b). Above the growth optimum, ingestion rates increased with increasing food C : P ratios but levelled off above food C : P ratios of 400 (Fig. 4a). Despite increased food ingestion rates, estimated P‐intake rates decreased strongly with increasing food C : P but slower than predicted by the 1 : 1 line (Fig. 4b). P‐loss rates were largely proportional to P‐intake rates (Fig. 5), with low loss rates under high C : P food and high loss rates when animals were fed low C : P food. The response of P‐loss rate to increasing P‐intake rate nevertheless tended to saturate at the lowest levels of food C : P (Fig. 5).

## Discussion

Our study clearly demonstrates the existence of a stoichiometric knife‐edge for somatic as well as population growth rates. Both variables showed a unimodal response along the food C : P gradient, with optimal growth at intermediate food C : P ratios and reduced growth rates towards the extremes of the food quality gradient. The results are well in line with studies reporting a stoichiometric knife‐edge for a variety of organism groups (Bullejos *et al.* 2014; Benstead *et al.* 2014; Laspoumaderes *et al.* 2015; Elser *et al.* 2016). In our study, the food C : P ratios at which somatic and population growth were found to be optimal were strikingly similar (i.e. approximately 170), although the slopes of the responses tended to be steeper for population than for somatic growth rate. Population growth rate integrates life history and demographic processes and may therefore be a more sensitive indicator of the adverse effects of stoichiometric mismatch than somatic growth rate. Indeed, stoichiometric mismatch is known to strongly affect life history traits such as survival, fecundity and development time (Jensen & Verschoor 2004; Felpeto & Hairston 2013; Zhou *et al.* 2018). The more pronounced negative response of population growth rate to stoichiometric mismatch likely reflects these additional effects.

In contrast to what is widely assumed for consumers in general, the elemental composition of rotifers showed a non‐linear response to the food stoichiometry gradient. Within a food C : P range between 53 and 391, rotifers proved to be strong regulators. Rotifer C : P increased with food C : P but the extent of this increase was limited, suggesting strong homeostasis (Persson *et al.* 2010). In contrast, when food C : P trespassed the value of 391, homeostasis broke down entirely as rotifer C : P increased proportionally with food C : P. These results demonstrate an important heterogeneity in the degree to which an organism is able to adhere to homeostasis. The evaluation of the homeostatic strength of a consumer is often based on a single value (regulation coefficient, H; Sterner & Elser 2002) assuming a constant response strength of consumer to producer elemental composition across the whole stoichiometric food quality spectrum. However, care should be taken with the use of this metric, especially when organisms are being compared that have grown under different ranges of food C : P (see also Persson *et al.* 2010; Meunier *et al.* 2014).

The simultaneous study of consumer growth performance, body elemental composition and key physiological responses along a 10‐fold food C : P gradient provides insights into a number of fundamental questions, such as which are the challenges organisms are confronted with along opposite sides of the food C : P optimum, and how and to what extent organisms are able to respond to these challenges. The approach also casts a light on whether deviations from elemental homeostasis reflect adaptive physiological responses or, alternatively, an inability to cope with stoichiometric mismatch. In an almost threefold food C : P range surrounding the growth optimum (120–320), maintenance of a relatively strong degree of homeostasis resulted in no considerable performance reductions. These results indicate that rotifers strive towards maintaining an optimal body elemental composition to safeguard optimal growth and that they are also well able to do so when facing food with quite pronounced but still moderate deviations of their food C : P optimum. In contrast, at lower food C : P values (< 75), we observed a strong reduction in performance despite a relatively strong maintenance of elemental homeostasis. This performance reduction likely reflects large costs associated with maintaining homeostasis. Contrary to the TER idea, costs associated with the avoidance and removal of P were likely more important than effects of C limitation, given that we worked at food satiating conditions, and that carbon is a very abundant element even in food with a very low C : P. Indeed, animals responded to lowering food C : P by reducing feeding and thus C‐ and P‐intake rates (Fig. 4) and by increasing P‐loss rates (Fig. 5; Frost *et al.* 2004, 2005; He & Wang 2007). Both strategies, however, come at costs and have their limitations. Reduced food uptake rates may secondarily result in a reduced acquisition of energy (Plath & Boersma 2001; Suzuki‐Ohno *et al.* 2012) and other resources. Excretion of excess P has also been suggested to be energetically costly (Anderson *et al.* 2005; Boersma & Elser 2006; Elser *et al.* 2006) and the relative decline of P loss at the lowest levels of food C : P is also suggestive of physiological limitations to the rates at which P may be excreted. Finally, although rotifers responded with a mere 14% relative increase in somatic P to an approximately twofold increase in P relative to C in their diet, this increase in somatic P may nevertheless have negatively impacted the performance of the rotifers through toxic effects (Karasov & Martinez del Rio 2007) or costs associated with its storage (Persson *et al.* 2010).

When food C : P exceeded 391, a complete breakdown of homeostasis (Fig. 2) coincided with a strong reduction in somatic and population growth rates (Fig. 3). This shows a strongly reduced ability of rotifers to cope with such high degree of stoichiometric mismatch and also demonstrates negative performance effects of deviations from an optimal body stoichiometry. Rotifers thus do not seem flexible conformers but regulators that have limited capacities to maintain homeostasis when confronted with high C : P food. Above the optimum, rotifers responded to an increasing food C : P with increased (‘compensatory’) feeding, a well‐documented response of consumers to compensate for the reduced intake of essential nutrients when facing nutrient deficient food (Fink & von Elert 2006; Suzuki‐Ohno *et al.* 2012). However, above a food C : P of 400, feeding rates levelled off, possibly due to a trade‐off between food‐ingestion and P‐assimilation rates (DeMott *et al.* 2010) and the need to avoid excess C intake (Hessen & Anderson 2008). Rotifers also responded to increased food C : P with a strong reduction of P‐loss rates, although they seemed unable to achieve a reduction of P loss stronger than proportional to what they acquired through feeding. The dramatic increase in rotifer C : P above the food C : P optimum tended to be more driven by a reduced P than an increased C content suggesting that homeostatic breakdown was mainly caused by the inability to cope with lack of P than excess carbon, although the latter also likely played a role.

Our results support the conclusions of Meunier *et al. *(2014) of nonlinearity in the response of consumer body stoichiometry to food elemental composition. Application of the framework of Meunier *et al. *(2014) to our results nevertheless remained difficult. According to Meunier *et al. *(2014), breakdown of homeostasis in regulators occurs at both sides of the food optimum when food C : P values surpass specific thresholds. Although we indeed observed such pattern at high food C : P, no breakdown of homeostasis was observed at the low food C : P end. This is most likely because the lowest C : P value of our experimental food quality range was still too high and we cannot exclude the possibility that a breakdown would still be observed at lower food C : P levels. It is nevertheless remarkable that rotifers were better able to maintain homeostasis when facing low compared with high C : P extremes. Indeed, in the range above the optimum, homeostatic breakdown was observed close after the food C : P ratio doubled compared to the optimal food C : P. In contrast, in the range below the optimal food C : P, homeostatic breakdown was not observed when the ratio of P to C in the food tripled. This asymmetry in response is likely a reflection of the fact that organisms are facing fundamentally different challenges along these opposite directions of the resource quality gradient. When faced with increasing C : P above the optimum, animals suffer from the simultaneous combination of P limitation and excess C in their food. In contrast, given that we worked at food satiating conditions, animals exposed to low C : P food only had to face excess P but no limited availability of C. The combined effect of excess C and limitation of P may therefore explain the faster breakdown of homeostasis in the higher than in the lower food C : P ranges.

### Relevance to natural systems

The strong negative effects of excess P on the performance of consumers may cast a new light on the consequences of eutrophication (Elser *et al.* 2016). In the latest decades, many water bodies worldwide have undergone strong eutrophication due to anthropogenic N and P inputs. These nutrient inputs and associated changes in light regimes (light‐nutrient hypothesis; Sterner *et al.* 1997; Elser *et al.* 2003) have strongly lowered C : nutrient ratios of primary producers in many of these systems. The low C : P levels applied in our study correspond well with the lower range of values reported for lake seston in surveys (Hessen 2006; Sterner *et al.* 2008). Indirect negative effects of eutrophication on primary zooplankton consumers, for example, via the promotion of toxic or inedible cyanobacterial blooms (Carpenter 2008; Schindler *et al.* 2008) or the enhancement of planktivorous fish stocks (Sereda *et al.* 2008) are quite well understood. Remarkably, much less attention has been given to the potential detrimental direct effects of stoichiometric mismatch caused by high levels of nutrient supply (Boersma & Elser 2006; Elser *et al.* 2016). The results of our study support the idea that the high P content in food seston itself may be an underappreciated factor that may not only contribute to the reduction of primary consumer performance in eutrophied systems (Elser *et al.* 2016) but that may also affect ecosystem functions, for example, by altering mass‐specific rates of grazing and P excretion.

As consumers of primary producers, recyclers of nutrients and food source of higher trophic levels, zooplankton take a key position in the pelagic food web. An important question is to what extent the observed heterogeneity in homeostatic strength impacts these functions along broad gradients of relative nutrient supply rates. Several models in ES describe consumer–producer and nutrient cycling dynamics assuming strict consumer homeostasis (Sterner 1990; Loladze *et al.,* 2000), whereas more recent models (Mulder & Bowden 2007; Anderson *et al.* 2013) suggest that a relaxation of homeostasis may change model outcomes. Another prominent question is to what extent homeostatic breakdown of primary consumers may result in a stoichiometric mismatch with higher trophic levels. P limitation of primary consumers has indeed been shown to travel up the food chain (Malzahn *et al.* 2007; Boersma *et al.* 2008), and subsequently affect energy transfer efficiencies among higher trophic levels (Grover 2003; Rowland *et al.* 2015). Recent studies have also shown that consumer prey with excess P may negatively affect the performance of their predators (Benstead *et al.* 2014; Laspoumaderes *et al.* 2015), and one may hypothesise that such bottom‐up cascades may also be driven by very high P supply under hypereutrophic conditions. However, given the limited response of rotifer P‐content and C : P ratios to P‐rich food, our results provide limited support for such mechanism.

Although our results were obtained in well‐defined laboratory conditions, they may form a poor representation of the complexity of real field conditions. Our results focus on the response of a single consumer to elemental mismatch with one producer. In natural systems, however, consumer communities consist of multiple species that may differ from each other in their responses to elemental mismatch (Currier & Elser 2017). Furthermore, consumers are exposed to a mixture of food source types that may vary in elemental composition and on which they may feed selectively. In addition, consumer populations may also be negatively affected by indirect, non‐stoichiometric effects of elemental imbalance in their environment (Rothhaupt 1995; Zhou *et al.* 2018). For example, algae grown in P‐limited environments have been shown to have a reduced digestibility (van Donk *et al.* 1997) or changed biochemical composition (Spijkerman & Wacker, 2011; Challagulla *et al.,* 2015). We are not aware of studies that have demonstrated such indirect effects for P‐rich environments, but the possibility that such effects further aggravate the consequences of excess P cannot be a *priori* excluded. Therefore, although our results provide strong proof of concept for a number of important ideas that so far have received limited attention in ES, there is a need for more studies to address their relevance to the complex situation of natural systems. With that respect, some studies have been recently done, for example, regarding the strength of the knife‐edge effect in multiple *Daphnia* species using lake seston (Currier & Elser 2017) or elemental plasticity at species and community level in outdoor mesocosms (Teurlincx *et al.* 2017).

## Authorship

LZ and SAJD developed the idea and designed the experiments. LZ carried out the experiments and performed the data analysis. LZ and SAJD wrote the manuscript.

## Data Availability

Data available from the Dryad Digital Repository: https://doi.org/10.5061/dryad.84q3757.
