# Supplementary material for: Herbivore consumers face different challenges along opposite sides of the stoichiometric knife‐edge
Source: Ecol Lett. 2019 Sep 11;22(12):2018–27. doi: 10.1111/ele.13386 (PMC6900088; doi:10.1111/ele.13386)
Supplement: Supplementary file 1 [file ELE-22-2018-s001.pdf]

Appendix S1

Algae cultures and food preparation

We used the green algae *C. reinhardtii* as food source for the rotifers. The algae were cultured in continuous 2L-chemostats using modified WC medium (Guillard and Lorenzen, 1972) at room temperature of 25 ˚C and a dilution rate of 0.33 day^{-1}. The WC medium contained 15 µmol L^{-1} P. Phytoplankton chemostats received ≈120 µmol quanta m^{-2} s^{-1} of continuous light. Algal biomass and stoichiometric composition were in a steady state at least for three months prior to the experiment (see Zhou et al. 2018).

To create food with different C:P ratios, we first harvested algae directly from the chemostat, and then manually added the inorganic phosphate (K_2HPO_4, 0.05 mol L^{-1}) 90 minutes before feeding them to experimental rotifers. The algae were then kept in a shaking incubator under darkness to ensure the adequate absorption of P and to avoid photosynthesis and growth of the algae (Zhou et al. 2018). The concentrations of algal food were calculated based on the equation: Y = 21.456 X (R^2=0.9899), where Y is the carbon concentration of algal food (µmol L^{-1}) whereas X represents the biovolume (10^6 μm^3) as measured from the coulter counter. This equation was also used for calculating the carbon concentrations in the food ingestion rate experiment.

Estimation of population growth rates

Before the experiment, we acclimatized nine rotifer populations to each of the food quality treatments for 10 days by initiating cultures of each food quality treatment in small scales (20 ml flasks) and expanding the cultures of each population. At the end of this period we transferred 4000 individuals into 200 mL flasks with a 1000 µmol C L^{-1} food suspension of the corresponding food quality treatments. To achieve continuous exponential growth we daily restarted these populations in a fresh food suspension with a subsample of approximately 800 individuals. At the end of each 24-hour period, we estimated total population size in each of the cultures by counting the number of rotifers in two independent 5 mL subsamples. Based on these counts a culture volume equivalent to 4000 rotifers was rinsed on a 60 µm mesh and used to restart the respective populations with fresh medium. All the flasks were incubated in a shaking incubator at 24 ˚C under continuous darkness. We repeated this culturing process during the whole experimental period (i.e. 10 days of acclimatization followed by 12 days of experiment).

Estimation of somatic growth rates

The somatic growth rate experiment aimed to study the response of somatic growth rate to the experimental C:P gradient. For each food quality treatment we kept a rotifer population in exponential growth (see 'population growth rate'). The experiment was started by transferring at least 400 mothers with parthenogenetic eggs form each culture into a fresh food suspension (1000 µmol C L-1) of the respective quality. After 2 hours, we incubated 100 newborns from these cultures in a glass vial with 5 ml of the corresponding food suspension, covered these vials with aluminium foil, and placed them on a rotating plankton-wheel (30 rotations per minute) to keep the algal food in suspension. After 18 hours, each cohort was prepared for the measurement of total C content (Mt). For each food quality treatment we also measured total carbon content for a group of 100 newborns with a maximum age of 2 hours (M0). Mass-specific somatic growth rate (h^{-1}) was calculated as (lnMt – lnM0)/t, with t = 18 hours, and subsequently converted to somatic growth rate at time interval of 24 hours (day^{-1}).

Estimation of food ingestion rates

To start the grazing experiment, we isolated three replicate groups of 200 rotifer individuals from each of the nine food quality treatments in the population growth rate experiment. To avoid variation due to rotifer age, sex or size, we selected exclusively adult females carrying one parthenogenetic egg. These groups of animals were incubated in 10 ml glass vials filled with 8 ml of food suspension (1000 µmol C L^{-1}) of the respective food quality treatments and allowed to feed for four hours. During the incubation, the glass vials were wrapped in aluminium foil to prevent growth of algae and placed on a rotating plankton wheel to keep algal food in suspension (30 rotations $minute^{-1}$). Immediately after the incubation period, all rotifers of each vial were retrieved and used to start the subsequent P loss experiment (see P loss experiment).

Appendix S2:

Appendix S2 Table 1 Results of quadratic linear regression models. Models in bold are those selected for interpretation based on AIC and consistency (see Methods).

Variables		AIC	df	F	p	R^2	Adjusted R^2	Equation
**Dependent variables**	**Explanatory variables**							
**Population growth rate**	**Diet C:P ratiosƚ**	**-33.9**	**$DF_{2,6}$**	**50.53**	**<0.001**	**0.944**	**0.925**	**$y=-4.439+1.395X - 0.094X^2$**
**Somatic growth rate**	**Diet C:P ratiosƚ**	**-23.6**	**$DF_{2,6}$**	**34.58**	**<0.001**	**0.920**	**0.894**	**$Y=-5.716+1.947X - 0.132X^2$**
**Food ingestion rate**	**Diet C:P ratios**	**2.7**	**$DF_{2,6}$**	**130**	**<0.001**	**0.977**	**0.970**	**$Y=2.125+0.0126X-0.0000105X^2$**
**P intake rateƚ**	**Diet C:P ratiosƚ**	**-186.73**	**$DF_{2,24}$**	**51.98**	**<0.001**	**0.813**	**0.797**	**$Y=0.241-0.0469X+0.0024X^2$**
**P loss rateƚ**	**P intake rateƚ**	**-16.2**	**$F_{2,6}$**	**233.6**	**<0.001**	**0.987**	**0.983**	**$Y=-7.33-0.740X-0.131X^2$**
Rotifer C:Pƚ	Diet C:P ratiosƚ	-10.2	$F_{2,6}$	33.27	<0.001	0.917	0.890	$Y=10.40-1.312X+0.103X^2$
Population growth rate	Rotifer C:P ratios	-16.1	$F_{2,6}$	4.36	=0.067	0.593	0.457	$Y=-0.513+0.024X -0.00013X^2$
Somatic growth rate	Rotifer C:P ratios	-10.4	$F_{2,6}$	5.69	=0.041	0.655	0.540	$Y=0.072+0.027X-0.00016X^2$
Rotifer C:N ratiosƚ	Diet C:P ratiosƚ	-21.0	$F_{2,6}$	20.08	=0.002	0.870	0.827	$Y=4.20-0.559X + 0.044X^2$

ƚ represents the values that have been Log2-transferred during the analysis.

Appendix S2 Table 2 Results of piecewise regression models. Models in bold are those selected for interpretation based on AIC and consistency (see Methods).

Variables		AIC	Equation 1	Equation 2	Breakpoint	Slope1	95% CI (slope 1)	Slope2	95% CI (slope 2)	p_{Davies}
**Dependent variables**	**Explanatory variables**									
Population growth rate	Diet C:P ratiosǂ	-36.5	Y=-0.4317+0.16X	Y=3.4-0.232X	200.9	0.16*	(0.09,0.23)	-0.39*	(-0.29,-0.18)	<0.001
Somatic growth rate	Diet C:P ratiosǂ	-22.5	Y=-0.113+0.215X	Y=4.098-0.338X	196.2	0.215*	(0.055,0.374)	-0.338*	(-0.461,-0.215)	0.004
Food ingestion rate	Diet C:P ratios	1.5	Y=2.54+0.007X	Y=8.47-0.005X	493.7	0.007*	(0.006,0.009)	-0.005	(-0.014,0.004)	0.023
P intake rateǂ	Diet C:P ratiosǂ	-184.8	Y=0.14-0.016X	Y=0.064-0.006X	187.4	-0.016*	(-0.03,-0.006)	-0.006	(-0.014,0.002)	0.212
P loss rateǂ	P intake rateǂ	NA	NA	NA	NA	NA	NA	NA	NA	NA
**Rotifer C:P ǂ**	**Diet C:P ratiosǂ**	**-20.04**	**Y=5.4665+0.1255*X**	**Y=-2.266+1.0235X**	**390.7**	**0.125***	**(0.05,0.20)**	**1.023***	**(0.58,1.47)**	**0.008**
**Population growth rate**	**Rotifer C:P ratios**	**-31.6**	**y = 1.79-0.031X**	**y=3.657-0.037X**	**80.1**	**0.031**	**(-0.06,0.13)**	**-0.037***	**(-0.007,-0.004)**	**0.004**
**Somatic growth rate**	**Rotifer C:P ratios**	**-27.2**	**Y=-5.355+0.0883X**	**Y=2.067-0.00825X**	**76.9**	**0.0883**	**(-0.031,0.208)**	**-0.00825***	**(-0.01,-0.006)**	**0.006**
**Rotifer C:N ratiosǂ**	**Diet C:P ratiosǂ**	**-24.0**	**Y=2.482-0.008X**	**Y=0.86+0.202X**	**183.2**	**-0.008**	**(-0. 18,0.16)**	**0.207***	**(0.07,0.33)**	**0.178**

*slope differing significantly from 0; p_{Davies}: significance of difference between the two slopes; ǂ Log2-transformed prior to statistical analysis. NA: no breakpoint detected.

Appendix S2 Table 3 Results of linear regression models. None of these models were selected for further interpretation given their low performance compared to quadratic or piecewise regression models.

**Variables**		**AIC**	**df**	**F**	**R^2**	**Adjusted R^2**	**p**	**Equation**
**Dependent variables**	**Explanatory variables**							
Population growth rate	Diet C:P ratiosǂ	-10.5	$DF_{(1,7)}$	0.377	0.05	-0.08	=0.56	Y=0.71-0.019X
Somatic growth rate	Diet C:P ratiosǂ	-4.0	$DF_{(1,7)}$	0.984	0.12	-0.002	=0.35	Y=1.54-0.043X
Food ingestion rate	Diet C:P ratios	1.5	$DF_{(1,7)}$	91.98	0.93	0.92	<0.001	Y=2.74+0.006X
P intake rateǂ	Diet C:P ratiosǂ	-185.4	$DF_{(1,25)}$	92.91	0.79	0.78	<0.001	Y=0.11-0.011X
P loss rateǂ	P intake rateǂ	-12.5	$DF_{(1,7)}$	287.3	0.98	0.97	<0.001	Y=-3.49+0.697X
Rotifer C:Pǂ	Diet C:P ratiosǂ	-4.49	$DF_{(1,7)}$	28.91	0.81	0.78	=0.001	Y=4.78+0.23X
Population growth rate	Rotifer C:P ratios	-14.0	$DF_{(1,7)}$	3.90	0.36	0.27	=0.089	y =0.84-0.003X
Somatic growth rate	Rotifer C:P ratios	-9.1	$DF_{(1,7)}$	7.15	0.51	0.43	=0.032	Y= 1.67-0.005X
Rotifer C:N ratiosǂ	Diet C:P ratiosǂ	-17.6	$DF_{(1,7)}$	22.61	0.764	0.730	=0.002	Y=1.81+0.098X

ǂ represents the values that have been Log2-transferred during the analysis.

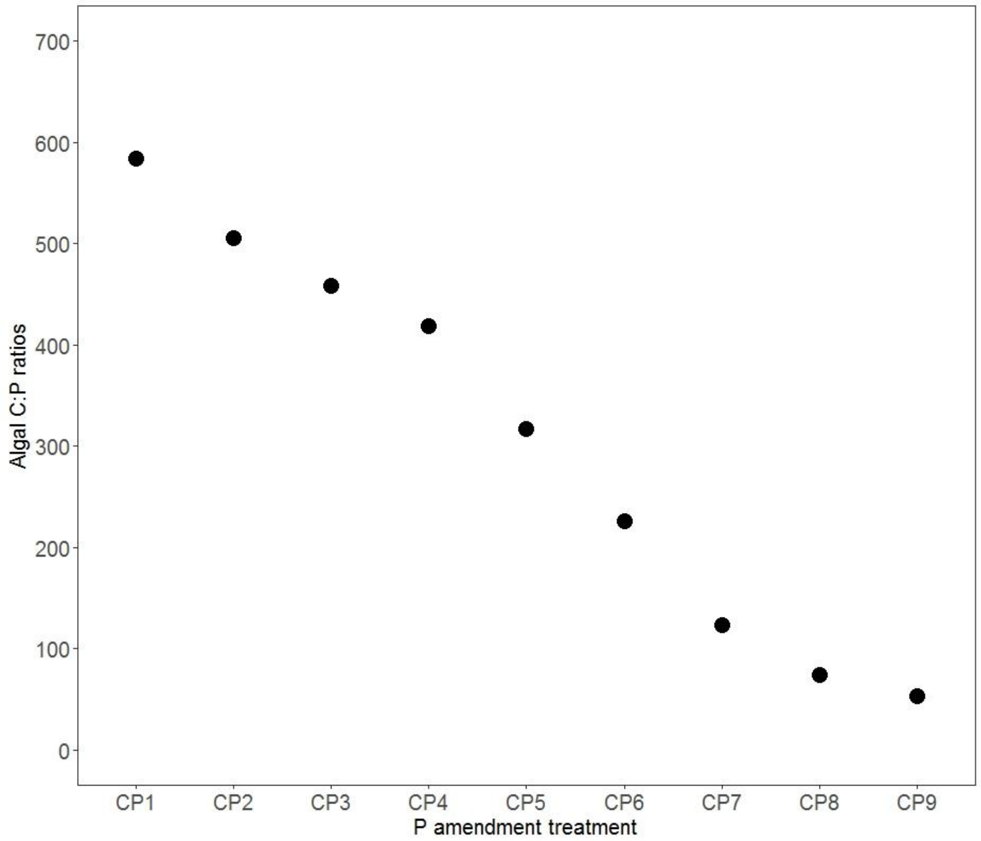

Appendix S2 Figure 1 Algal molar C:P ratios of each food quality treatment. Symbols are the mean values of three repeated measurements in time.

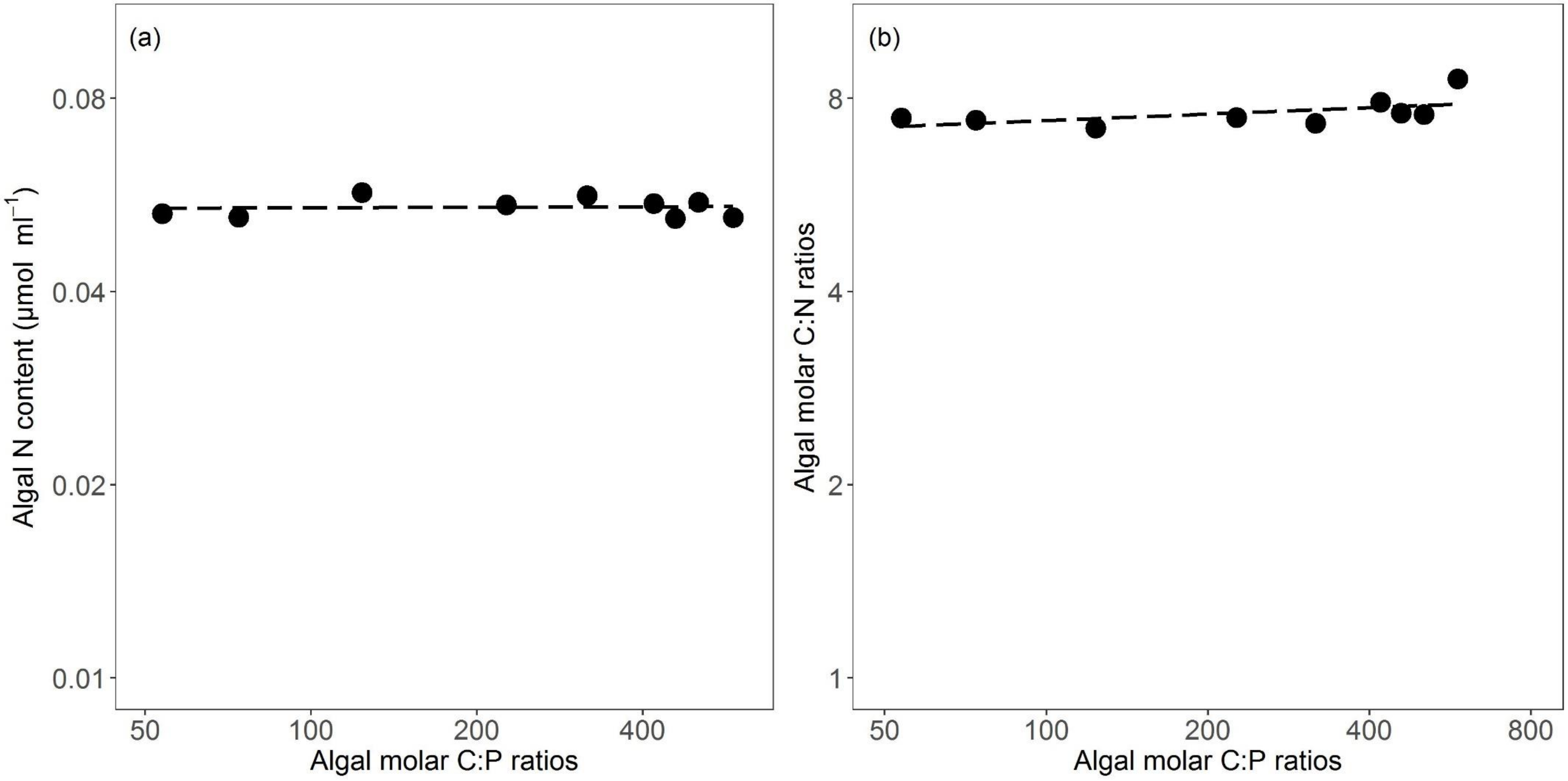

Appendix S2 Figure 2 (a-b) Algal N content (a) and C:N ratios (b) of each food quality treatment. Linear regression models showed a slope of 0.0024 for algal N content which was not significantly different from 0 (p=0.88), and a slope of 0.032 for algal C:N ratios in response to algal C:P ratios (p=0.128). Symbols are the mean values across three repeated measurements in time. Note the log-scale of the axes.

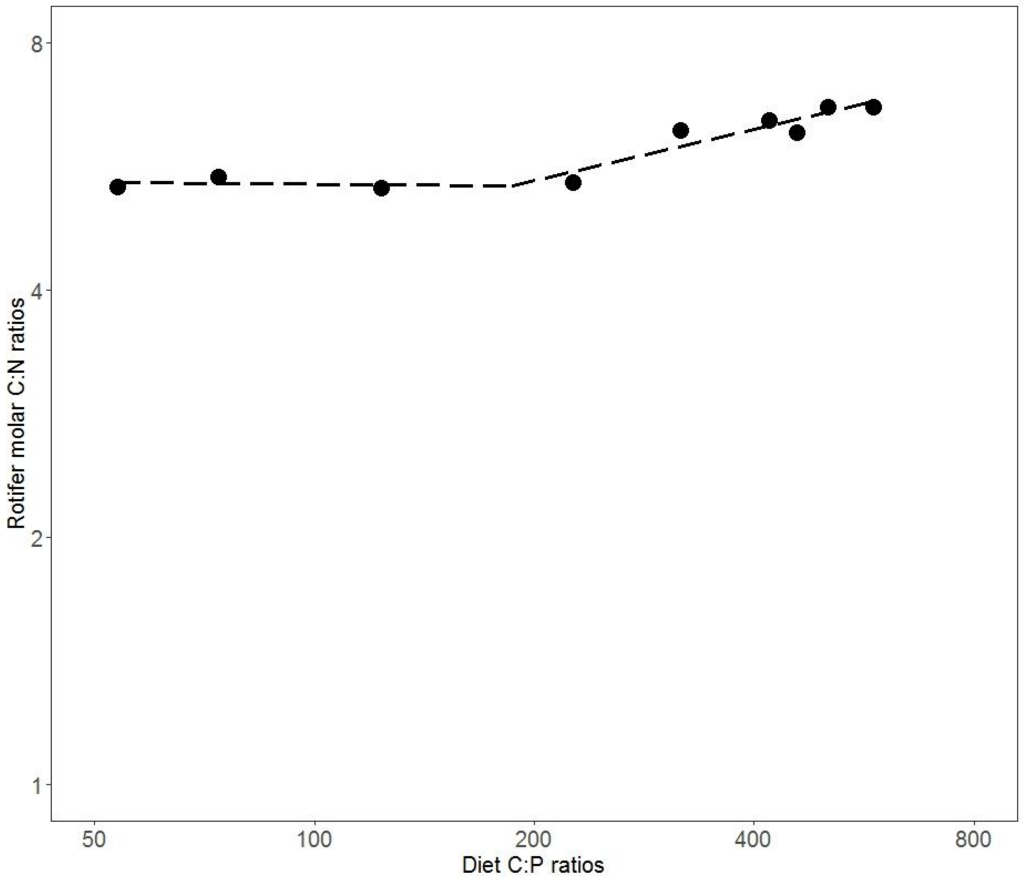

Appendix S2 Figure 3 Rotifer C:N ratios in each of the food quality treatments. The segmented regression shows a break point located at an algal C:P ratio of 186. The slope of the regression equaled -0.01 below and 0.21 above this breakpoint. These slopes did not differ significantly (Davies-test). Symbols represent the mean values across three repeated measurements in time. Note the log-scale of the axes.

Results for Rotifer C and P analysis

At the breakpoint food C:P of 391, rotifer C:P equaled 93.5. At the highest food C:P level (584), rotifer C:P equaled 137. Above the breakpoint, rotifer C:P thus responded with a 46% increase to a 50% augmentation of the C:P of its food. Such dramatic increase of rotifer C:P appears to have resulted from the joint effects of increased total somatic C content and reduced total somatic P content. Indeed, for rotifer C content, a two segments piecewise regression model suggested a breakpoint at a food C:P of 418. The slope of the regression above this breakpoint was steeper than the slope of the regression below this breakpoint (0.485 and 0.042, respectively; Figure 2b), although no significant difference between these two slopes was detected (p=0.259; Appendix S2 Table 4). For rotifer P content, a breakpoint occurred at a food C:P of 312. With increasing food C:P the rate at which rotifer P content decreased was larger above than below this breakpoint (p = 0.019; see Appendix S2 Table4).

To assess the relative contribution of changes in somatic C and P content to variation in rotifer C:P ratio, we calculated rotifer C:P ratios keeping one element (either C or P) constant while allowing the other one to vary. At the highest food C:P level assuming an invariable rotifer P, the observed increase in C content would result in a rotifer C:P of 107, corresponding to a 19% increase. With an unchanged rotifer C content, the decrease in P content would result in a rotifer C:P of 134, corresponding to a 43% increase. These results suggest that the breakdown of homeostasis at high food C:P ratios was mainly driven by reductions of body P content, although augmentations in C content also had a considerable contribution.

Appendix S2 Table 4 Results of piecewise regression models for rotifer C and P contents

**Variables**		**AIC**	**Equation 1**	**Equation 2**	**Breakpoint**	**Slope1**	**95% CI (slope 1)**	**Slope2**	**95% CI (slope 2)**	**p_{Davies}**
**Dependent variables**	**Explanatory variables**									
Roitfer C content	Diet C:P ratios	-16.5	Y=0.042X + 4.087	Y=0.485X + 0.231	417.6	0.042	(-0.047,0.131)	0.485	(-0. 055,1.024)	0.259
Rotifer P content	Diet C:P ratios	-26.4	Y=-0.07X - 1.46	Y=-0.491X + 2.074	335.2	-0.07*	(-0.121,-0.0186)	-0.491*	(-0.801,-0.181)	0.008

*slope differing significantly from 0; p value: significance of difference between the two slopes (p<0.05 represent a significant difference); data was log2-transferred before analysis.
